# Liver’s influence on the brain through the action of bile acids

**DOI:** 10.3389/fnins.2023.1123967

**Published:** 2023-02-02

**Authors:** Xin Yi Yeo, Li Yang Tan, Woo Ri Chae, Dong-Yup Lee, Yong-An Lee, Torsten Wuestefeld, Sangyong Jung

**Affiliations:** ^1^Institute of Molecular and Cell Biology (IMCB), Agency for Science, Technology and Research (A*STAR), Singapore, Singapore; ^2^Department of Psychological Medicine, Yong Loo Lin School of Medicine, National University of Singapore, Singapore, Singapore; ^3^Department of BioNano Technology, Gachon University, Seongnam, South Korea; ^4^School of Chemical Engineering, Sungkyunkwan University, Suwon, South Korea; ^5^Genome Institute of Singapore (GIS), Agency for Science, Technology and Research (A*STAR), Singapore, Singapore; ^6^School of Biological Sciences, Nanyang Technological University, Singapore, Siingapore; ^7^National Cancer Centre Singapore, Singapore, Singapore; ^8^Department of Physiology, Yong Loo Lin School of Medicine, National University of Singapore, Singapore, Singapore

**Keywords:** liver, brain, bile acid, neurodegeneration, gut microbiome

## Abstract

The liver partakes as a sensor and effector of peripheral metabolic changes and a regulator of systemic blood and nutrient circulation. As such, abnormalities arising from liver dysfunction can influence the brain in multiple ways, owing to direct and indirect bilateral communication between the liver and the brain. Interestingly, altered bile acid composition resulting from perturbed liver cholesterol metabolism influences systemic inflammatory responses, blood-brain barrier permeability, and neuron synaptic functions. Furthermore, bile acids produced by specific bacterial species may provide a causal link between dysregulated gut flora and neurodegenerative disease pathology through the gut-brain axis. This review will cover the role of bile acids—an often-overlooked category of active metabolites—in the development of neurological disorders associated with neurodegeneration. Further studies into bile acid signaling in the brain may provide insights into novel treatments against neurological disorders.

## 1. Introduction

The liver is the largest internal solid organ in the human body with responsibility for various vital roles, such as macronutrient metabolism and homeostasis (Rui, 2014; Alves-Bezerra and Cohen, 2017; Kraft et al., 2017), regulation of endocrine signaling (Bikle, 2021), blood volume (Eipel, 2010), and the detoxification of xenobiotics (Grant, 1991; Björkholm et al., 2009). As a result of its proximity and the presence of blood vasculature and biliary system directly connecting the liver to the gut and the heart, the liver is strategically positioned to pick up toxic compounds, sense and respond to metabolic changes through the production of paracrine and endocrine signals (Rhyu and Yu, 2021). Furthermore, the liver and brain are interconnected by the parasympathetic and sympathetic nervous systems, allowing for bidirectional communication and further control of liver and body homeostasis by the central nervous system (CNS). While the afferent fiber senses and convey changes in the portal vein blood composition to the central nervous system, the penetration of splanchnic and vagal nerve sympathetic fibers into the liver parenchyma and the hepatic lobules (Fukuda et al., 1996; Jensen et al., 2013) and the presence of cell-cell gap junctions allow efficient transmission of feedback autonomic and hormonal signals to control liver function (Kandilis et al., 2015).

Due to the potential impact of the liver filtrates and products on multiple organ systems (Hadjihambi et al., 2018; Herrero et al., 2020), conditions leading to hepatic failure can be debilitating and life-threatening and require early diagnosis and careful management (Squires et al., 2018; Ginès et al., 2021; Søreide and Deshpande, 2021). Interestingly, patients with chronic liver disease or liver failure often develop neurological complications (Louissaint et al., 2022), suggesting the breach of non-effectively removed toxins or normal peripheral-restricted liver metabolic byproducts with direct effects in the nervous system (McMillin et al., 2016) or the liver-dependent dysregulation of the immune system affecting the neurological outcome (Sureka et al., 2015). Specifically, dysregulated bile acid (BA) metabolism and levels are associated with the rapid deterioration of neurological function in various neurological diseases (Crick et al., 2017; Chen et al., 2019), as opposed to observed neuroprotective functions of the BAs (Moreira et al., 2017; Graham et al., 2018; Thams et al., 2019).

**FIGURE 1:**
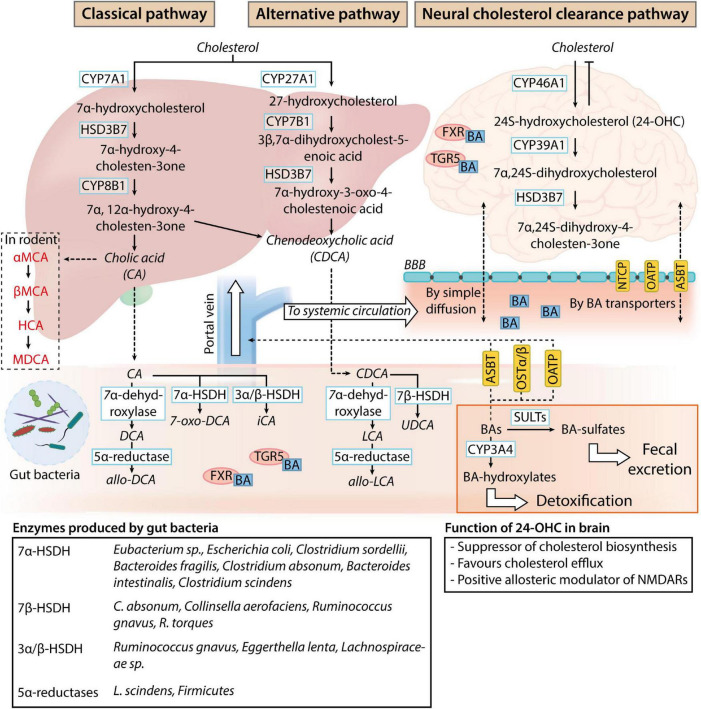
Bile acid metabolism and circulation. In normal physiological conditions, the classical pathway accounts for 90% of the bile acids (BAs) present in the human body. While BAs are produced as systemic modulators of physiological processes by the liver (classical and alternative pathways), sterols are produced as by-products of the brain cholesterol clearance process (neural cholesterol clearance pathway). Primary BAs produced by the liver are deposited in the gallbladder and released into the intestine upon stimulation by the hunger hormone cholecystokinin for lipid emulsification. Once in the gastrointestinal tract, primary BAs are readily deconjugated, dehydroxylated, or dehydrogenated by gut microbes. The main bacterial enzymes involved in the process are listed in the table at the bottom of the image. These modified BAs (secondary BAs) can proceed to exert their effects as a negative modulator of gut function through bile acid receptor pathways [Takeda G-protein-coupled receptor 5 (TGR5) and farnesoid X receptor (FXR) signaling] or gut microbiome composition. After which, BAs can be passively reabsorbed into the liver for further conjugation with taurine or glycine and enter the systemic circulation and the central nervous system through simple diffusion or BA transporters present on the blood-brain barrier (NTCP, sodium taurocholate co-transport peptide; OATP, organic anion transporting polypeptides; ASBT, apical sodium bile acid transporter). 24-hydroxycholesterol (24-OHC) produced by the neural cholesterol clearance pathway negatively regulates cholesterol metabolism in the brain, while the physiological function of systemic BAs in the nervous system is unclear. BAs can be transported back into the intestine (ASBT, OATP, and OSTα/β, organic solute transporter α, and β) and further sulfated by sulfotransferase (SULT) or hydroxylated by Cytochrome P450, family 2, subfamily a, polypeptide 4 (CYP3A4) for detoxification or clearance through fecal excretion.

Bile acids are physiological detergent-like amphipathic agents synthesized from cholesterol through a complex biosynthesis process managed by 17 enzymes of Cytochrome P450s (CYPs) in the liver hepatocytes (Figure 1). Although the neutral (classical) and the acidic (alternative) pathways are responsible for human primary BA production (Chiang, 2013; Baloni et al., 2020), most of the total BAs pool synthesis occurs through the classical pathway. The classic synthesis pathway is initiated by the rate-limiting enzyme cholesterol 7α-hydroxylase (CYP7A1) in the endoplasmic reticulum of the hepatocytes. CYP7A1 hydroxylates the 7α-position on cholesterol to produce 7α-hydroxycholesterol, which is further processed to give 7α-hydroxy-4 cholesten-3-one (C4) by 3β-hydroxy-Δ5-C27-steroid dehydrogenase/isomerase (HSD3B7). C4 is then metabolized into cholic acid (CA) and chenodeoxycholic acid (CDCA) in the presence and the absence of 12α-hydroxylase (CYP8B1), respectively (Baloni et al., 2020).

The alternative pathway synthesizes the remaining 10% of BAs, which consists predominantly of CDCA (Smith et al., 2009). In the alternative pathway, 27-hydroxycholesterol and 3β-hydroxy-5-cholestenoic acid are sequentially produced with sterol 27-hydroxylase (CYP27A1)-dependent oxidation of cholesterol. The 3β-hydroxy-5-cholestenoic acid is then converted to 3β,7α-dihydroxycholest-5-enoic acid by 7α-hydroxylase (CYP7B1). As the liver is the sole organ possessing all enzymes required for bile acid synthesis, the oxidized sterols generated by the alternative pathway need to be transported back to the liver for final conversion into CDCA (Chiang, 1998; Pandak and Kakiyama, 2019). Oxidized sterol intermediates formed in peripheral tissues expressing CYP27A1 and CYP7B1 are converted to CDCA through the alternative pathway. Although the classical pathway contributes to most of the bile acid production in physiological conditions in humans, the alternative pathway predominates as the major bile acid synthesis pathway in the presence of liver diseases (Chiang, 1998; Vaz and Ferdinandusse, 2017).

Before the excretion of primary BAs from the liver, conjugation with either taurine or glycine occurs in hepatocytes. These conjugated BAs are also referred to as bile salts and possess increased water solubility and lower cytotoxicity for subsequent function in the intestines (Baloni et al., 2020; Grant and DeMorrow, 2020). CA and CDCA conjugation with glycine forms glycocholic acid (GCA) and glycochenodeoxycholic acid (GCDCA), while conjugation with taurine forms taurocholic acids (TCA) and taurochenodeoxycholic acid (TCDCA), respectively (Grant and DeMorrow, 2020). The conjugated bile acids are excreted across the hepatocyte’s apical membrane into the bile capillaries and deposited in the gallbladder. Upon food consumption, the hunger hormone cholecystokinin released by enterocytes from the brush border of the small intestines triggers the parasympathetic stimulation of splanchnic and vagal nerve (Kanno et al., 2001; Chiang, 2013), causing the release of bile salts in the biliary tract into the duodenum. In the intestines, most of the primary BAs are actively reabsorbed in the ileum.

Unabsorbed primary BAs are modified into secondary BAs by gut microbes through deconjugation, dehydroxylation, and dehydrogenation processes (Guzior and Quinn, 2021). Bile salt hydrolases (BSH) that are responsible for the enzymatic hydrolysis of C-24 N-acyl amide bond and removal of glycine or taurine conjugates from BAs are widely expressed in the human gut microbiome (Jones et al., 2008; Song et al., 2019). Although the significance of BA deconjugation is not fully understood, it is thought that BSH is required for the detoxification of BAs that function as bacteria toxins (Grill et al., 2000; Dussurget et al., 2002) and restricts pathogen colonization of the gastrointestinal (GI) tract (Allain et al., 2018). Further biotransformation of primary to secondary BAs is dependent on the hydroxysteroid dehydrogenases (HSDH) and dehydroxylases found in *Clostridiaceae* (Vital et al., 2019), *Bacteroides* (Macdonald et al., 1975; Fukiya et al., 2009), *Eubacterium* (MacDonald et al., 1977; Baron et al., 1991), *Eggerthella* (Mythen et al., 2018), and *Lachnospiraceae* spp (Vital et al., 2019), along with individual bacteria species such as *Ruminococcus gnavus* and *Escherichia coli* (Tanaka et al., 1996). Deoxycholic acid (DCA), ursodeoxycholic acid (UDCA), and lithocholic acid (LCA) can be passively reabsorbed and further conjugated with taurine or glycine in the liver (Trauner and Boyer, 2003; Thomas et al., 2008).

Classically, BAs play a pivotal role in emulsifying fat globules for further digestion by lipases to form glycerol and fatty acids. 95% of the BA released is subsequently reabsorbed by intestinal enterocytes of the upper intestinal tract through passive diffusion. They can be taken up *via* active transport by the sodium-dependent bile acid transporter (ASBT) in the ileum enterocytes (Mertens et al., 2017) or by membrane transporters sodium taurocholate cotransporting polypeptide (NTCP) and organic anion transporter polypeptide (OATP) (Grant and DeMorrow, 2020), and are transported back to the liver through the hepatic portal vein. The addition of glucuronic acid to BA by uridine 5’-diphosphate-glucuronosyltransferase (UGT) aids BA detoxification and increases their hydrophilicity to encourage urinary excrement (Pauli-Magnus and Meier, 2005). *De novo* liver BA synthesis accounts for 5% of the BA lost in fecal excretion (Chiang, 2013). Despite the potential impact of BAs on human cognition, there has been a lack of understanding of the contribution of BA to neuronal function and pathophysiology. In this review, we summarize the current observed roles of BAs in normal neurologifcal physiology and examine their involvement in the development of neurodegenerative conditions. Through examination of the novel role of BAs in neurological function and disorders, we aim to provide an alternate perspective on the environmental contribution toward neurological dysfunction.

## 2. The effects of bile acids on normal neurological function and neurological-dependent modulation of metabolic physiology

### 2.1. Impact of bile acids on neurological function under physiological conditions

In the nervous system, the neural cholesterol clearance pathway has been suggested to participate in the synthesis of CDCA. Although transcriptomic analysis of post-mortem brain samples revealed the expression of the CYP27A1 and CYP7B1 genes, there is an absence of CYP7A1 and CYP8B1 protein expression (Baloni et al., 2020), driving the production of BAs in the brain through the alternative pathway or the neural cholesterol clearance pathway *via* CYP46A1 expressed in the brain. 24S-hydroxycholesterol produced from cholesterol in the brain has an increased solubility which allows for its passage across the blood-brain barrier (BBB) into systemic circulation *via* the ATP-binding cassette transporter 1 (ABCA1) (Dietschy, 2009). The sterol intermediate eventually gets converted into 7α,24S-dihydroxycholesterol, followed by 7α,24S-dihydroxy-4-cholesten-3one by CYP39A1 and HSD3B7, respectively, in the liver (McMillin and DeMorrow, 2016).

While the gut-specific effects of BA are frequently studied, the role of BA in the nervous system is relatively less understood. Unconjugated and conjugated BAs can enter the CNS through different mechanisms and the amount of BAs in the cerebrospinal fluid correlates to their plasma concentrations (Higashi et al., 2017). Conjugated acids, which are structurally larger and amphipathic are transported through the blood-brain barrier (BBB) with the apical sodium-dependent bile acid transporter (ASBT), organic anion-transporting polypeptide (OATP), or sodium taurocholate co-transporting polypeptide (NTCP) (Mertens et al., 2017). Unconjugated BAs such as CA, CDCA, and DCA can passively diffuse across the BBB. Interestingly, the gut-dominant BA receptors such as the GpBAR1, nuclear receptor FXR (Mertens et al., 2017), glucocorticoid receptors (GR) (Muzzi et al., 2021), the pregnane X receptor (PXR) (Litwa et al., 2016), vitamin D receptor (VDR) (Eyles et al., 2005, 2014), sphingosine-1-phosphate receptor 2 (S1PR2) (McMillin and DeMorrow, 2016; McMillin et al., 2017) and muscarine acetylcholine receptors (M3) (Poulin et al., 2010) are present in the nervous system, suggesting a role for BAs in regulating neurological function.

Regions of the nervous system situated outside the BBB are particularly susceptible to the effects of BAs. The effects of BAs are prominent in patients suffering from chronic liver conditions, who develop pruritus from the activation of MAS Related GPR Family Member X4 (MRGPRX4) receptor expressed on dorsal root ganglion histamine receptor H1 (HRH1)-positive neurons by BA that have escaped the liver (Yu et al., 2019). In normal physiology, BAs are found to rapidly accumulate in the hypothalamus and suppress food intake through the recruitment of the GpBAR1 pathway (Perino et al., 2021). The anorexigenic effect is likely due to BA-GpBAR1-dependent inhibition of the agouti-related protein/neuropeptide Y (AgRP/NPY) in the arcuate nucleus (ARC). Concomitantly, intraperitoneal administration of FGF15/19 commonly produced from BA signaling in the gastrointestinal tract can evoke FGFR-ERK1/2 activity in the ARC AgRP/NPY neurons (Marcelin et al., 2014). DCA can further signal satiety through the cholecystokinin type A (CCK-A) and GpBAR1 expressing nodose ganglia neurons, to the hypothalamic nucleus to reduce food intake (Wu et al., 2020). Beyond these rudimentary understandings of the role of BA in neurological function, little is known about their contribution to complex central homeostatic regulation.

Further examination of BA signaling in the CNS suggests broad BA-dependent neurological effects. Global FXR ablation increased serum BA levels and altered BA composition in the brain. The change affected numerous neurotransmitter systems in the CNS, leading to impaired memory and motor coordination (Huang et al., 2015). This is consistent with the role of BA as a direct antagonist of several neurotransmitter receptors within the CNS–muscarinic acetylcholine receptors (Raufman et al., 2002), the γ-aminobutyric acid type A (GABA_A_) receptors, and the N-methyl-D-aspartate (NMDA) receptors (Kiriyama and Nochi, 2019). In addition, serum bile acid levels can affect respiratory rhythm through the FXR in the brainstem medulla (Zhao et al., 2014). BAs are potential neuroimmunological modulators. Lithocholic acid (LCA), DCA, and CDCA activation of GpBAR1 induces the production of reactive oxygen and nitrogen species in primary astrocytes (Keitel et al., 2010) while reducing microglial activation (Chiang and Ferrell, 2019). While FXR is normally expressed at low levels in the neurons in the primary cortex, its expression is upregulated upon neuroinflammation (Shan et al., 2020). The stimulation of the neuronal and astrocyte-localized LCA receptor VDR with physiological levels of an active metabolite of Vitamin D reduces calcium channel expression and protects hippocampal neurons against excitotoxicity (Brewer et al., 2001). Interestingly, maternal intrahepatic cholestasis or overnutrition is associated with altered metabolism and hepatic function in the offspring (Papacleovoulou et al., 2013; Thompson et al., 2022), with an increased risk of developing cognitive deficits and neuropsychiatric disorders (Sullivan et al., 2012; Wolfrum and Peleg-Raibstein, 2019). S1PR signaling is implicated in neuronal differentiation (Lee et al., 2011), neurite outgrowth during development (Yang et al., 2020), and the determination of neuronal properties (Li et al., 2015). The potential role of BA in neurological development may provide clues to neurodevelopmental deficits linked to metabolic disturbances during pregnancy.

### 2.2. Bile acid as a signaling moiety and regulator of metabolic homeostasis

In addition to their physiological detergent role, BAs serve as a feedback regulator of metabolic homeostasis by regulating metabolic gene expression within the gastrointestinal tract. The conjugated and unconjugated forms of DCA and LCA function as agonists of FXR (Sun et al., 2021) which induce fibroblast growth factor 15/19 (FGF15/19) production. FGF15/19 activates fibroblast growth factor receptor (FGFR4) in hepatocytes, leading to the downregulation of CYP7A1 mRNA expression and BA production (Kong et al., 2012). In the enterocytes, FXR activation inhibits the sodium-dependent bile acid transporter (SLC10A2) while recruiting the bile acid binding protein (BABP) and the organic solute transporter α and β heterodimer (OSTα and OSTβ) to reduce BA reabsorption and increase hepatocyte secretion of BA into portal blood (Chiang and Ferrell, 2020). Alternate nuclear receptors such as the hepatocyte nuclear factor 4α (HNF4α) (Inoue et al., 2006) and liver X receptor α/β (LXRα/β) (Peet et al., 1998) have also been linked indirectly to the BA-dependent regulation of BA pool composition. Alternatively, DCA, CDCA, TCA, TDCA, and TUDCA can activate the insulin signaling Ak strain transforming (AKT) pathway through Gαi protein-dependent receptors or mitochondria generation of superoxide to mimic the effect of insulin, which likely enhance insulin function between feeding cycles (Han et al., 2004; Dent et al., 2005; Fang et al., 2007). The small heterodimer partner (SHP)-FXR complex also plays a role in the control of glucose and lipid metabolism (Calkin and Tontonoz, 2012).

BAs can further fine-tune energy homeostasis *via* modulation of food and energy processing. Within brown adipose tissues, CA increases energy expenditure by activating the intracellular thyroid hormone-activating enzyme type 2 iodothyronine deiodinase (D2) (Ockenga et al., 2012). G protein-coupled bile acid receptor 1 (GpBAR1) is a heterotrimeric Gs-coupled receptor expressed on a subpopulation of myenteric neurons within the intestine and acts as a molecular switch in the presence of deoxycholic acid (DCA) to regulate the contractile activity of the colonic tissue (Poole et al., 2010). The receptor is accessible by BA under normal physiological conditions, as the BAs are rapidly deconjugated and made more permeable to the mucosa by the gut microbiome (Molinero et al., 2019). As such, the systemic activation of GpBAR1 by notoginsenoside Ft1 alleviates metabolic alternations observed in diet-induced obesity mice (Ding et al., 2021). Concurrently, the presence of BA exerts a strong selective pressure on the gut microbiome composition, leading to a preferential enhancement of bacteria capable of BA catabolism and a reduction in populations with prominent anti-obesogenic effects (Zheng et al., 2017), which may explain the correlation between gut microbiome dysbiosis and metabolic dysfunction observed in obesity patients (Xu et al., 2022).

## 3. Contribution of bile acid and its signaling components to neurological pathophysiology

Liver dysfunction is often associated with an altered neurological function (Leung and Young, 2018). In particular, individuals suffering from liver diseases such as chronic liver damage and liver cirrhosis are predisposed to encephalopathy (Newell, 1984; Felipo, 2013; Ferro and Oliveira, 2014). In the event of liver dysfunction, the amount of BAs present in the systemic circulation could increase up to 100 times the physiological level. Due to their amphipathic and detergent-like properties, BAs can lead to modification, at lower quantities, or damage lipid layers of BBB at high quantities (Naqvi et al., 1970; Greenwood et al., 1991). In obstructive cholestasis, the elevation of serum DCA and CDCA likely trigger Rac1 activation in vascular endothelial cells, leading to the increase in permeability of the BBB (Quinn et al., 2014), consequently allowing the same molecules or toxins to enter the brain (Bron et al., 1977). The current understanding of hepatic encephalopathy (HE) revolves around the concept of hyperammonemia-dependent astrocytic swelling and brain edema resulting from hyperammonemia (Butterworth, 2013; Elwir and Rahimi, 2017). Given the interplay between BA and immune system function and the longstanding relationship between liver disease and neuroinflammation (Butterworth, 2013), with the ability of BA or cholesterol sequestrant in preventing and alleviating the neurological complications of HE (McMillin et al., 2016, 2018), aberrant BA signaling is a likely contributor to HE.

BAs possess potent antimicrobial activities that shape the gut microbiome composition (Tian et al., 2020). Through direct interaction with their native receptors present within the gastrointestinal tract, BAs can activate interleukin 18 (IL-18), inducible NO synthase (iNOS), and carbonic anhydrase 12 (CAR12) involved in the inhibition of bacteria overgrowth and enteric protection (Inagaki et al., 2006). Alternatively, BAs exert cytotoxic effects on individual bacteria species—*Bacteroides* (Binder et al., 1975), *Clostridia* (Binder et al., 1975), *Lactobacillus* (Kurdi et al., 2006), *Bifidobacterium* (Kurdi et al., 2006), *Escherichia coli* (Sung et al., 1993), *Enterococcus fecalis* (Sung et al., 1993), likely through their amphipathic properties that disrupt the bacterial cell membrane, damage the proteins and genetic materials required for normal cellular function (Urdaneta and Casadesús, 2017). CA can inhibit the growth of Gram-negative bacteria while potentiating the abundance of *Clostridium XIVa* and 7α-dehydroxylase expressing bacteria (Winston and Theriot, 2020; Yang et al., 2021). On the other hand, DCA inhibits Gram-positive bacteria (Xu et al., 2021). It is not clear how the preferential antimicrobial effect is achieved by the BAs. The effects of BA are particularly evident in the human small intestine suffering from a bacteria overgrowth problem due to the reduction in BA secretion during bile duct litigation or liver cirrhosis (Slocum et al., 1992; Ding et al., 1993). This is opposed to the normal small intestine that contains high amounts of conjugated BA and a relatively low number of microbes (Gorbach et al., 1967). To combat the deleterious effects of BA, bacteria have evolved mechanisms to deal with and prosper in the BA-enriched environment. The efflux system encoded by the acrAB and emrAB genes is important for the expulsion of bile from the bacteria cell (Ma et al., 1994, 1995; Lomovskaya et al., 1995). Alternative, undiscovered energy-dependent systems likely co-exist alongside the acrAB/emrAB system to help in BA efflux since de-energized *Escherichia coli* carrying acrA and emrB mutations strongly accumulate CDCA within the bacteria compartments (Thanassi et al., 1997). DC and CDCA enhanced *Shigella* adherence to the surrounding tissue and invasion capabilities by inducing Ipa protein secretion (Pope et al., 1995). As such, long term-supplementation with exogenous single BAs can induce gut dysbiosis and imbalance in BA metabolism (Xu et al., 2021).

Studies conducted on patients with different neurological diseases often report an alteration in plasma or cranial BA levels, which correlates with peripheral BA levels. Metabolomic studies in post-mortem Alzheimer’s disease (AD) brain samples presented an enhanced brain-specific CYP27A1 activity with a relatively higher ratio of TCA, DCA, LCA, TDCA, and GDCA (Baloni et al., 2020). Taurine transport is also upregulated in AD brains, which suggests a possible enhanced taurine-conjugation of CDCA and other subspecies. Further analysis of the AD-associated BA composition revealed a correlation between the extent of BA dysregulation and gut microbiome activity (MahmoudianDehkordi et al., 2019). Similarly, an increase in plasma levels of unconjugated BA is observed in human Parkinson’s disease (PD) patients and murine PD models (Yakhine-Diop et al., 2020). Although there is no evidence of overgrowth of *C. scindens* responsible for the heightened levels of serum LCA and DCA observed in the A30P α-synuclein PD mouse model (Li et al., 2020), individuals suffering from *C. difficile* infection have a significantly heightened short-term risk of contracting PD (Kang et al., 2020). This is consistent with the observation that an enhancement in secondary bile acid production following cholecystectomy is linked to an increased risk of developing PD (Kim et al., 2021). Interestingly, the primary BA CA is effective in preventing Aβ-42 plaque formation (Majid et al., 2019) while TUDCA exerts context-dependent anti-apoptotic and anti-inflammatory effects (Keene et al., 2002; Rodrigues et al., 2002), suggesting a difference in the neurological effect of different BA species. TUDCA activates GpBAR1, and inhibits the pro-inflammatory NFκB pathway in microglia, resulting in the inactivation of proinflammatory status in multiple sclerosis (MS) (Bhargava et al., 2020). Despite a lack of consensus if BA disturbance is a response toward developing neuropathology or the initial cause of the neuropathology observed (Dodge et al., 2021), the alteration of BAs content in the plasma can serve as an early diagnostic biomarker for AD (Shao et al., 2020), PD (Graham et al., 2018), MS (Bhargava et al., 2020), and amyotrophic lateral sclerosis (ALS) (Nakazato et al., 2022).

Neuroinflammation and reactive gliosis is an important contributor of the pathology of neurodegenerative conditions (Pekny and Pekna, 2016; Kwon and Koh, 2020). Endogenous levels of TUDCA blocks *in vitro* neurotoxic polarization of astrocytes and microglia induced by IFN-γ and LPS (Bhargava et al., 2020). This is likely due to the recruitment of the GpBAR1 pathway and pyruvate kinase 2 (PKM2) that regulates the NLR family pyrin domain containing 3 (NRLP3) inflammasome activity (Islam et al., 2022; Romero-Ramírez et al., 2022). Furthermore, dendritic cells (DC) from mice fed with a DCA- but not a CA-enriched diet reduced DC polarization toward the T helper (Th) 1/17 axis and response toward immunological priming (Hu et al., 2021). It must be noted that the involvement of interleukin 17 (Fu et al., 2022) and resident brain and lymphatic DC (Greter et al., 2005; Bossù et al., 2015) in human neurodegeneration is controversial and the contribution of BA toward the modulation of neurodegeneration through the DC/Th17 pathway requires in-depth examination. Alternatively, BA has been shown to induce the production of pro-inflammatory cytokines and chemokines independent of the potential cytotoxic effects of BA on hepatocytes, which recruit and retain neutrophils in the liver (Allen et al., 2011). The release of reactive oxygen species (ROS) by recruited neutrophil damage and kills hepatocytes (Gujral et al., 2004), with time driving the development of HE. Consistent with the involvement of BA in BBB permeability, high levels of cytokine in systemic circulation can cause a breach in the BBB, allowing the infiltration of peripheral immune cells and liver products into the brain (Kempuraj et al., 2017).

## 4. Discussion

Despite an extensive understanding of the role of general BA metabolism and BA signaling on gastrointestinal function, only a handful of reports have examined their distribution, metabolism, and contribution to the nervous system function. Given the similarity in the structure of BAs (Fiorucci et al., 2018), the pleiotropic effects of BA, and the relatively indiscriminate activation of BA receptor by any of the BAs present in the system, the direct significance of altered BA levels or ratios in defined neurodegenerative conditions is elusive. While reports examining the differential contribution of BA physical properties in liver diseases suggest varying degrees of toxicity with BA hydrophilicity (Araki et al., 2001; Palmela et al., 2015), the relevance to their neurological function is unknown.

The mutual regulation between the gut microbiome and BA synthesis and function complicates the role of BAs in general physiology. Gut microbiota is known to modify host-derived BA into secondary BAs that exhibit alterations in patients with neuropsychiatric disorders (Aizawa et al., 2019; Liu et al., 2021; Zhang et al., 2022), while the BA composition can be affected by environmental stressors (Silvennoinen et al., 2015; Qu et al., 2022). The relative abundance of intestinal microbiota at the phylum level is modifiable by BA present within the system (Islam et al., 2011) through the direct antimicrobial effect of BA or BA effects on alternate antimicrobial molecules (Begley et al., 2005; Inagaki et al., 2006). As such, the cognitive capability of an individual is distinct and cannot be defined by understanding a single biological target or function of BA.

Bile acids (BA) can independently modulate the function and effects of the brain (Nunes et al., 2012; Bazzari et al., 2019), gut (Zhou et al., 2020), and gut microbiome (van Best et al., 2020), while each of these layers of the brain-gut-microbiome axis may contribute to the overall outcome in the brain (Garcia et al., 2022; Lirong et al., 2022; MahmoudianDehkordi et al., 2022; Qu et al., 2022). Hence, there is a large spectrum and significant complexity in how BA and the intestinal flora affect brain function. While current work examines the correlation between specified BA or bacterial targets and the changes in neurological outcomes, it is still a work in progress to dissociate their role and independent effects on brain functions.

Similarities in BA signaling pathways in both the gastrointestinal tissues and the brain suggest the potential for repurposing BA-related therapeutics for neurodegenerative conditions. Treatment of general health ailments with BA and its constituents has been a long-standing tradition in traditional Chinese medicine (Hagey et al., 1993; Feng et al., 2009). There has been extensive use of chemically synthesized BA and synthetic ligands of the FXR to target BA signaling pathways in liver diseases and metabolic conditions (Li and Chiang, 2014; Duan et al., 2022). The tolerability of specific BA supplementation in the human system (Ashby et al., 2018) and the success of TUDCA as a disease modifier in Stage II clinical trial targeting ALS (Paganoni et al., 2022) highlight the potential of BA for neurodegenerative conditions management. Nonetheless, extensive investigation of the effects of long-term modulation of BA pathways in normal cognitive functions is essential to ascertain the suitability of preexisting drugs designed for peripheral use in neurological treatment.

Technical limitations significantly slowed the progress of bile acid and gut microbiome research. Detection of BA content in biological samples through commonly adopted enzymatic assays and mass spectrometric methods has restricted resolution toward BAs with closely related structural features and BAs with abundance levels that differ significantly from the majority of the BA species in the sample (Dutta et al., 2019). Similarly, current microbiome research is over-reliant on non-representative fecal samples and metagenomics, with high dependence on pre-established gene catalogs or an understanding of microbiome characteristics and functions (Hooks et al., 2019). As such, conclusions are currently descriptive at best.

Examination of the unique aliphatic nature of BAs and known BA transporters present on the BBB provides valuable insights and alternative approaches to drug development for neurological conditions. The majority of existing drugs are insufficiently soluble and permeable for sufficient absorption into systemic circulation upon oral administration (Gupta et al., 2013). BA conjugated to lipid nanocarriers improves the absorption of packaged drug components in the gastrointestinal tract (Zhang et al., 2013; Yang et al., 2019), through ASBT-dependent transport of the drug-conjugated bile acids (Al-Hilal et al., 2014) or BA-dependent enhancement of drug amphipathic properties to improve cell permeation. Also, the conjugation of bile acids to drug molecules prolonged the drug’s half-life in circulation (Heinen et al., 2013; Alam et al., 2014). Once they have successfully entered systemic circulation, DCA and CDCA present within the drug formulation can interact directly with the BBB to disrupt tight junction proteins and increase the ease of transport of previously BBB impermeable molecules (Quinn et al., 2014).

## Author contributions

XY, LT, and SJ: conceptualization. XY, LT, WC, D-YL, and SJ: writing—original draft preparation. XY, Y-AL, TW, SJ, and D-YL: writing—review and editing. TW and SJ: supervision and funding acquisition. TW, SJ, and Y-AL: critical revision of the manuscript. All authors read and agreed to the published version of the manuscript.
